# Daily‐Life, Sensor‐Derived Tremor Measures Are Sensitive to Progression in Early Parkinson's Disease

**DOI:** 10.1002/ana.78236

**Published:** 2026-05-12

**Authors:** Nienke A. Timmermans, Ioan Gabriel Bucur, Diogo C. Soriano, Erik Post, Hayriye Cagnan, Sooyoon Shin, Max A. Little, Yordan P. Raykov, Bastiaan R. Bloem, Rick C. Helmich, Luc J.W. Evers

**Affiliations:** ^1^ Center of Expertise for Parkinson and Movement Disorders, Department of Neurology Donders Institute for Brain, Cognition and Behavior Radboud University Medical Center Nijmegen The Netherlands; ^2^ Institute for Computing and Information Sciences, Radboud University Nijmegen The Netherlands; ^3^ Federal University of ABC São Bernado do Campo Brazil; ^4^ Department of Bioengineering Imperial College London London UK; ^5^ Verily Life Sciences Dallas TX USA; ^6^ School of Computer Science, University of Birmingham Birmingham UK; ^7^ School of Mathematical Sciences, University of Nottingham Nottingham UK

## Abstract

**Objective:**

Sensitive outcome measures are critical for evaluating the efficacy of novel treatments for Parkinson's disease (PD). In this study, we assess the sensitivity to change of sensor‐derived daily‐life tremor measures over 2 years in unmedicated and medicated persons with early PD.

**Methods:**

We used 2‐year continuous wrist sensor data (median wear time: 22 hours/day) from the Personalized Parkinson Project (n = 462 medicated; n = 78 unmedicated at baseline), in combination with annual clinical evaluations of tremor severity. From the gyroscope data, we derived previously validated weekly measures for tremor time and power, which were smoothed over time using piecewise linear trend estimation. One‐ and 2‐year standardized response means (SRMs) were computed to compare the sensitivity to change between the sensor‐derived tremor measures and clinical tremor scores.

**Results:**

In unmedicated participants with tremor, sensor‐derived tremor measures demonstrated a high sensitivity to progression (2‐year SRMs ranged from 0.67 to 1.09), which was significantly larger than clinical tremor scores (2‐year SRMs ranged from 0.21 to 0.41). In medicated participants, sensor‐derived tremor time decreased (2‐year SRM of −0.18), which was associated with both an increase in dopaminergic medication dose and higher disease duration. In contrast, the sensor‐derived tremor power measures and clinical rest tremor scores (measured in the OFF state) increased slightly (2‐year SRMs ranging from 0.11 to 0.27).

**Interpretation:**

Before initiation of symptomatic treatment, sensor‐derived daily‐life tremor measures are substantially more sensitive to progression than clinical tremor scores, making them a promising tool to evaluate the efficacy of disease‐modifying treatments in early PD. ANN NEUROL 2026;100:282–294

As the prevalence of Parkinson's disease (PD) continues to increase,^1^ disease‐modifying or more effective symptomatic treatments are highly warranted to alleviate the burden of this chronic and progressive disease. However, adequate measures of clinical disease progression are still lacking, making it difficult to evaluate the effectiveness of novel treatments.^2^, ^3^ The currently used Movement Disorder Society‐Unified Parkinson's Disease Rating Scale (MDS‐UPDRS) depends on episodic and subjective assessments, limiting its sensitivity to measure disease progression.^4^ Wearable sensors that continuously and objectively assess PD symptoms in natural environments could address these issues.^5^, ^6^, ^7^, ^8^

In addition to being sensitive to change, digital outcome measures should capture concepts that are meaningful to patients.^9^, ^10^ In early PD, tremor is experienced as one of the most bothersome symptoms.^11^, ^12^, ^13^ Tremor not only interferes with daily activities, but has sensory and psychosocial impacts as well.^14^ It is influenced by emotional and cognitive stress, voluntary movements, and timing of treatments, making it a highly fluctuating symptom.^15^ This complicates its evaluation during the typically brief, episodic in‐person clinical visits.^11^

Earlier studies have shown that PD tremor can be measured accurately with wrist‐worn sensors.^16^ However, longitudinal studies evaluating tremor progression in daily‐life are scarce, with only 1 study with a 1‐year follow‐up.^7^ Therefore, the ability of sensor‐derived tremor measures to capture tremor progression remains largely unknown. Furthermore, previous studies investigating tremor progression using MDS‐UPDRS assessments have yielded conflicting results in varying study settings. PD tremor could increase, remain stable, or decrease over time.^17^, ^18^, ^19^, ^20^ Investigating tremor progression in naturalistic environments could improve our understanding of tremor pathophysiology.

In this study, we examined tremor progression over 2 years using continuous free‐living sensor data from 540 early‐stage PD participants from the Personalized Parkinson Project (PPP).^21^, ^22^ We have previously developed and validated an open‐source algorithm that enables reliable monitoring of PD tremor in real‐life conditions.^23^ In this study, we compare the sensitivity to progression of the sensor‐derived measures against clinical tremor observations and patient‐reported tremor severity based on the MDS‐UPDRS. To isolate the effects of disease progression and symptomatic medication, we separately analyze participants who remained unmedicated throughout the study follow‐up and those receiving symptomatic drug therapy. Additionally, we assess the effect of changes in dopaminergic medication dose on sensor‐derived tremor measures. Collectively, this study aims to lay the foundation for using real‐life, objective tremor outcomes to measure tremor progression, both in clinical trials evaluating disease‐modifying therapies and in cohort studies aiming to increase our understanding of tremor pathophysiology.

## Methods

### Study Design and Participants

**FIGURE 1:**
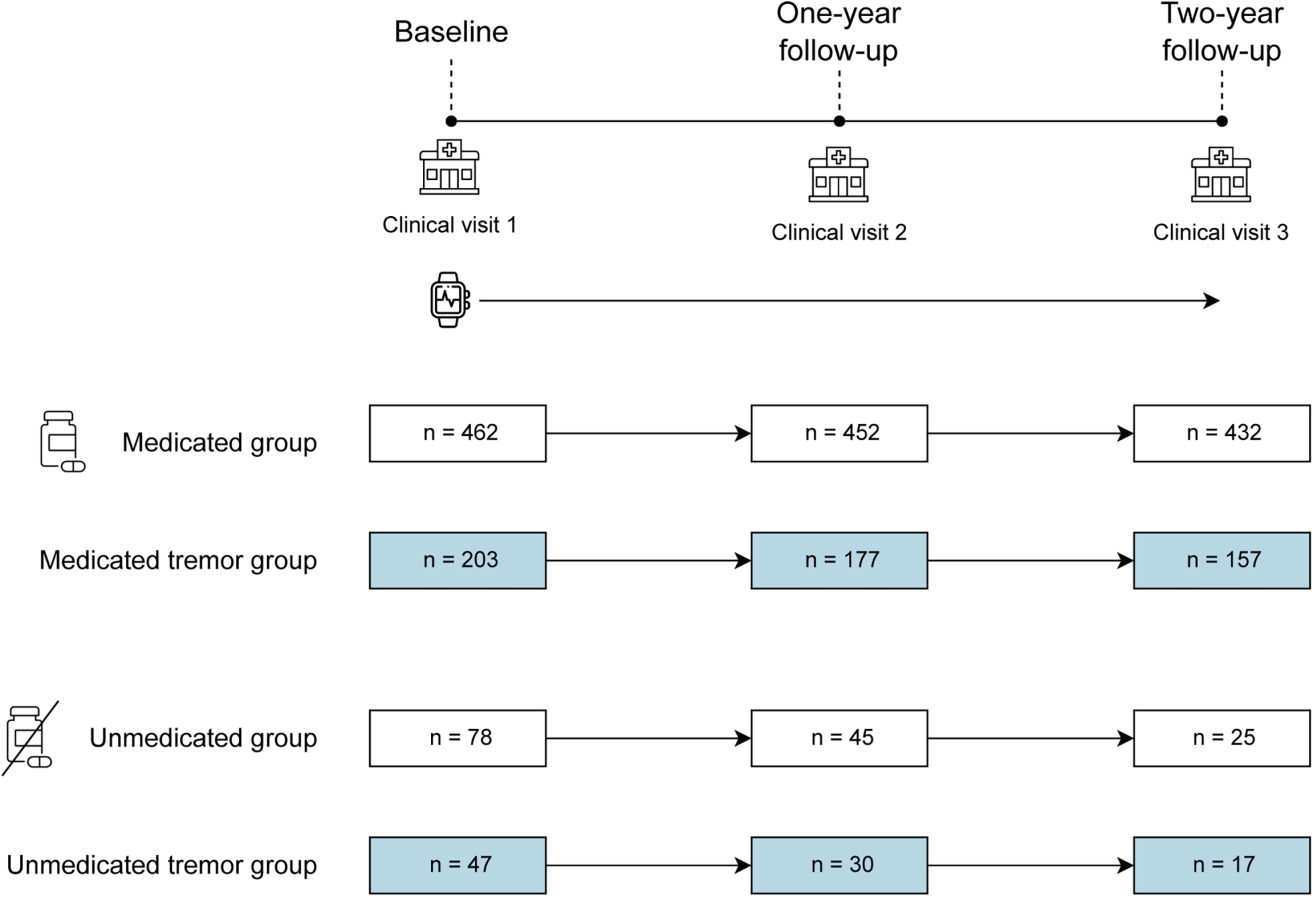
Schematic overview of the study design of the Personalized Parkinson Project and the subgroups used in this study. The tremor groups contain participants for whom the amount of detected tremor exceeded the false positive threshold at baseline and at follow‐ups. The number of included participants decreased over time because of dropout, initiation of symptomatic drug treatment (in the unmedicated groups), or disappearance of tremor (in de tremor groups). The subgroups used in our primary analysis focusing on long‐term tremor progression are highlighted in blue. [Color figure can be viewed at www.annalsofneurology.org]

Data were obtained from the PPP cohort and the PPP de novo cohort.^21^, ^22^ The PPP cohort included 520 subjects with early PD (disease duration ≤5 years). In the PPP De Novo cohort, 103 recently diagnosed (disease duration ≤2 years) and treatment‐naïve PD patients were included, who were not expected to start treatment in the first year after inclusion. In both cohorts, participants were monitored continuously for 2 years using the Verily Study Watch (median wear time of 22 hours/day^24^), collecting raw gyroscope, accelerometer, and photoplethysmography data. In addition, in‐clinic MDS‐UPDRS assessments were performed at baseline and after 1 and 2 years (see Fig 1).

Participants wore the watch on their preferred side, to maximize compliance. We excluded participants that switched their watch side during follow‐up (n = 25), and participants with fewer than 8 weeks of sensor data across the 2‐year follow‐up period (n = 8). Other exclusion criteria were having an alternative diagnosis (n = 15), starting dopaminergic medication within the first 8 weeks (n = 14), or having an unknown start date of dopaminergic medication (n = 21). This resulted in the inclusion of 540 participants with PD.

Because of the effects of symptomatic medication on tremor, for our primary analysis focusing on long‐term tremor progression we divided participants into: (1) a medicated group with participants who had already initiated symptomatic drug treatment at baseline; and (2) an unmedicated group, consisting of participants who were untreated at the time of inclusion. The number of participants in the unmedicated group mainly decreased over time because of initiation of treatment during the follow‐up period (Fig S1). Within these 2 groups, we created additional subgroups only including participants with tremor. This was based on whether the amount of watch‐detected tremor exceeded the false positive threshold of 3.5% tremor time, which was the 90th percentile of tremor time in controls.^23^ Together, this resulted in 4 analysis groups: medicated, medicated tremor, unmedicated, and unmedicated tremor, with group sizes varying across follow‐up (Fig 1).

The PPP studies were conducted in accordance with the Declaration of Helsinki and Good Clinical Practice guidelines and were approved by the local medical ethics committee (Commissie Mensgebonden Onderzoek, regio Arnhem‐Nijmegen, reference number 2016–2934 for PPP and 2020‐6,111 for PPP de novo). All participants provided informed consent before enrollment.

### Sensor‐Derived Tremor Measures

The continuous, high frequency (ie, 100Hz) gyroscope data collected by the watch was processed using the ParaDigMa toolbox,^25^ which contains a previously validated algorithm to detect and quantify PD rest and re‐emergent tremor.^23^ Three weekly aggregated tremor measures were derived: tremor time, modal tremor power, and the 90th percentile of tremor power. Tremor time was calculated as the percentage of inactive daytime with detected tremor. The modal tremor power captures the typical tremor severity, whereas the 90th percentile of tremor power represents the maximal tremor severity. To reduce computational load and data volume, we derived the weekly tremor measures every other week. Furthermore, the tremor power measures were only derived for weeks in which the amount of detected tremor time exceeded the false positive threshold, to avoid tremor power estimates in weeks where the majority of detected tremor likely reflected false positives. Further details of the tremor detection algorithm are described elsewhere.^23^

To focus on long‐term tremor progression rather than short‐term fluctuations or measurement error, we used piecewise linear trend estimation (L1‐trend filtering) to smooth the biweekly tremor measures over the 2‐year measurement period.^26^, ^27^ The trend was obtained by minimizing the following function:

(1) $$L\left(x\right)=\frac{1}{2}\sum_{t=1}^{n}{\left(y_{t}-x_{t}\right)}^{2}+\lambda \sum_{t=2}^{n-1}\left|x_{t-1}-2x_{t}+x_{t+1}\right|$$

where $y_{t}$ is the weekly tremor measure and $x_{t}$ is the fitted trend at time *t*. For each tremor measure, we determined the optimal smoothing parameter λ by selecting the value that yielded the best predictive accuracy of randomly masked observations across participants (ie, minimizing the mean absolute residual error across all participants, using leave‐one‐point‐out cross‐validation). The optimal λ and the proportion of variance explained by the fitted trends per measure are given in Table S1. In case of missing data, we interpolated and extrapolated the fitted trend for all weeks for which there was a tremor measure available in the surrounding 4 weeks. The resulting trends were used for all subsequent analyses.

### Sensitivity to Tremor Progression

We defined tremor progression as long‐term changes in tremor time and power, irrespective of their underlying cause (eg, neurodegeneration, symptomatic treatment, or behavior). The sensitivity to tremor progression of sensor‐derived measures was assessed in the medicated and unmedicated groups separately, and compared to the sensitivity of MDS‐UPDRS rest tremor scores and patient‐reported tremor score (items 3.17, 3.18, and 2.10), using 1‐ and 2‐year standardized response means (SRMs). An SRM of 0.2–0.5 indicates small responsiveness, 0.5–0.8 moderate responsiveness, and >0.8 large responsiveness.^28^ Ninety‐five percent confidence intervals (95% CI) were constructed using 10,000 bootstrap samples. Week 2 of the study period was selected as the baseline week to exclude potential effects of the baseline study visit. For the 2‐year follow‐up measures, week 100 was chosen to maximize the sample size available. Similarly, week 50 was used as the 1‐year follow‐up week. Clinical data were only included if the visit date was at 50 ± 10 weeks from baseline for the second visit, and at 100 ± 20 weeks from baseline for the third visit (Fig S2 shows the week number distribution for the third visit). Before assessing the SRM of tremor time, we transformed this measure using the logit function to approximate normality.

### Adjustment for Censoring

To assess tremor progression without the effects of symptomatic medication, we selected participants that remained untreated during the 1‐ or 2‐year follow‐up period. Although the decision to start treatment is likely influenced by multiple factors, specifically the effect of tremor severity on this decision could have introduced bias because of informative censoring, since our outcome was tremor severity over time. To ensure that this potential source of bias did not affect our results, we applied inverse probability‐of‐censoring weighting (IPCW), which reweighs remaining uncensored participants to reflect the composition of the cohort at baseline.^29^ We estimated the effect of tremor on the probability of censoring because of treatment initiation by fitting a Cox proportional hazards model in participants that were unmedicated at baseline (n = 78). Tremor time was used as the time‐varying covariate,^30^ averaged over 2 measurement weeks (the beta coefficient is shown in Table S2). We subsequently used this model to estimate the probability of each participant to remain unmedicated at each follow‐up week. The inverse probabilities were then used to reweigh participants within the unmedicated group for each week. To assess whether censoring of participants because of dropout could have affected our results, we compared the baseline characteristics of all medicated participants that were excluded from the 2‐year longitudinal analysis to those of the full baseline medicated group.

### Sensitivity Analyses

To assess the robustness of our findings, we performed several sensitivity analyses. First, we assessed the effect of follow‐up duration and smoothing on the observed SRMs, by visualizing SRMs over time based on smoothed and unsmoothed data. Second, we studied the effect of IPCW on the results by comparing the weighted and unweighted SRMs in the unmedicated group. Third, to assess the difference in tremor progression between the medicated and unmedicated groups while excluding potential effects of age, disease duration, and watch position (whether the watch was worn on the more‐ or less‐affected side^31^), we matched these subgroups at baseline using propensity score matching. We estimated propensity scores using logistic regression and matched participants 1:1 using nearest‐neighbor matching without replacement. The more‐ or less‐affected side was based on the sum of MDS‐UPDRS III tremor scores on both sides (4 items) in the OFF state. If those scores were equal, the patient‐reported more‐affected side was selected. If the participant also reported equally affected sides, we chose the side with the larger total MDS‐UPDRS III OFF score. Finally, we assessed whether the amount of sensor‐derived tremor time was related to watch‐sided hand dominance.

### Correlation with Changes in Patient‐Reported Tremor Score

To assess how objectively measured tremor changes relate to experienced tremor burden, we correlated the 2‐year changes in sensor‐derived tremor measures to the changes in the patient‐reported tremor score (MDS‐UPDRS 2.10). For comparison, we also assessed how changes in the clinical evaluation of rest tremor (MDS‐UPDRS 3.17 and 3.18) correlate to the patient‐reported tremor score.

### Effect of Changes in Dopaminergic Medication Dose

To study the effect of changes in dopaminergic medication dose on daily‐life tremor measures, we performed 2 additional analyses. First, we assessed the relation between 2‐year sensor‐derived tremor progression and changes in levodopa equivalent daily dose (LEDD), corrected for disease duration at baseline and watch position (more‐ or less‐affected side), by using multivariable linear regression in the medicated group. Second, we assessed the responsiveness of the sensor‐derived tremor measures to the initiation of dopaminergic treatment, using participants that initiated dopaminergic medication during the study period (n = 49). We calculated the absolute changes between the week before initiation of treatment and each week in a period of 0 to 14 weeks since the initiation of treatment, and we derived SRMs from these changes.

## Results

Table 1 shows the demographic and clinical characteristics, as well as sensor‐derived tremor measures of the medicated and unmedicated group at baseline.

**TABLE 1:** Demographic and Clinical Characteristics and Sensor‐Derived Tremor Measures at Baseline of the Medicated and Unmedicated Groups at Baseline

	Medicated group (n = 462)	Medicated tremor group (n = 203)	Unmedicated group (n = 78)	Unmedicated tremor group (n = 47)
Demographic and clinical characteristics at baseline				
Age (yr), median (IQR)	62 (56–69)	61 (55–67)	65 (56–70)	66 (56–71)
Gender (M), n (%)	273 (59)	131 (65)	48 (62)	28 (60)
Disease duration (mo), median (IQR)	33 (18–47)	38 (18–49)	5 (2–13)	8 (3–16)
Watch worn on more‐affected side, n (%)	246 (53)	131 (65)	60 (78)	39 (85)
MDS‐UPDRS part 1, median (IQR)	9 (7–14)	9 (7–14)	5 (3–7)	4 (3–7)
MDS‐UPDRS part 2, median (IQR)	7 (4–12)	7 (4–12)	4 (3–8)	4 (3–6)
MDS‐UPDRS part 3 OFF, median (IQR)	32 (24–42)	35 (26–45)	34 (28–40)	36 (29–42)
MDS‐ UPDRS part 4, median (IQR)	2 (0–5)	2 (0–5)	0 (0–1)	0 (0–3)
MDS‐UPDRS part 3 OFF tremor subscore, median (IQR)	4 (1–7)	7 (4–10)	5 (2–9)	7 (4–11)
Rest tremor severity device‐sided arm, median (IQR)	0 (0–1)	1 (0–1)	1 (0–2)	1 (1–2)
LEDD, median (IQR)	500 (300–688)^a^	460 (300–700)^b^	–	–
Sensor‐derived tremor measures at baseline				
Tremor time (%), median (IQR)	2.7 (1.2–8.4)	10.1 (5.5–25)	5.8 (1.4–43.1)	30.8 (10.7–60.2)
Modal tremor power (log values), median (IQR)	–	0.3 (0.2–0.7)	–	0.7 (0.2–1.2)
90th percentile of tremor power (log values), median (IQR)	–	1.6 (1.3–2.1)	–	1.9 (1.5–2.3)

The modal and 90th percentile of tremor power were assessed in all participants with tremor time above the false positive threshold at baseline (n = 203 for the medicated group and n = 47 for the unmedicated group). Part 1: non‐motor experiences of daily living. Part 2: motor experiences of daily living. Part 3: motor examination. Part 4: motor complications.

^a^ LEDD information of 20 participants was incomplete and 13 participants were using non‐dopaminergic medication.

^b^ LEDD information of 12 participants was incomplete and 9 participants were using non‐dopaminergic medication.

IQR = interquartile range; LEDD = levodopa equivalent daily dose; M = male; MDS‐UPDRS = Movement Disorder Society‐Unified Parkinson's Disease Rating Scale; mo = month; yr = years.

### Sensitivity to Tremor Progression

**FIGURE 2:**
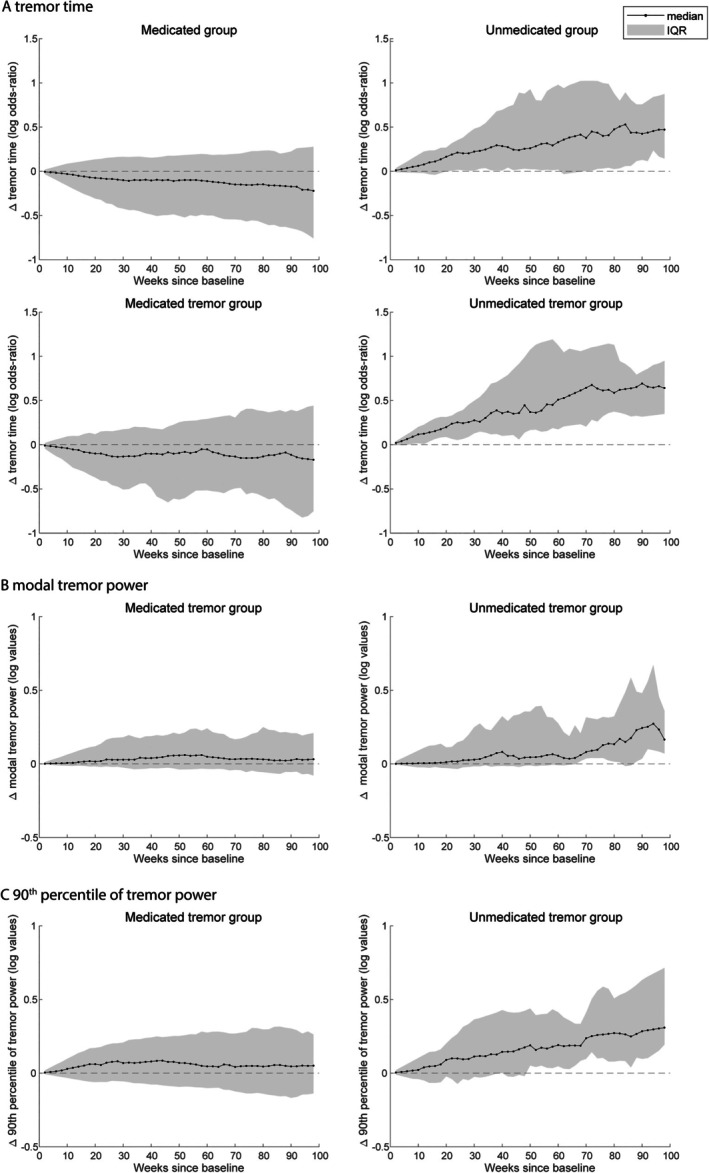
Median (and interquartile range [IQR] of) absolute within‐subject change since baseline of sensor‐derived weekly tremor measures, observed in the medicated (on the left) and unmedicated group (on the right). Tremor time (A) was derived for all participants for whom data was available at baseline and at follow‐up (upper plots), and subsequently for all participants with tremor time above the false positive threshold at baseline and at follow‐up (lower plots). The modal (B) and 90th percentile of tremor power (C) were also assessed for all participants with tremor time above the false positive threshold.

**FIGURE 3:**
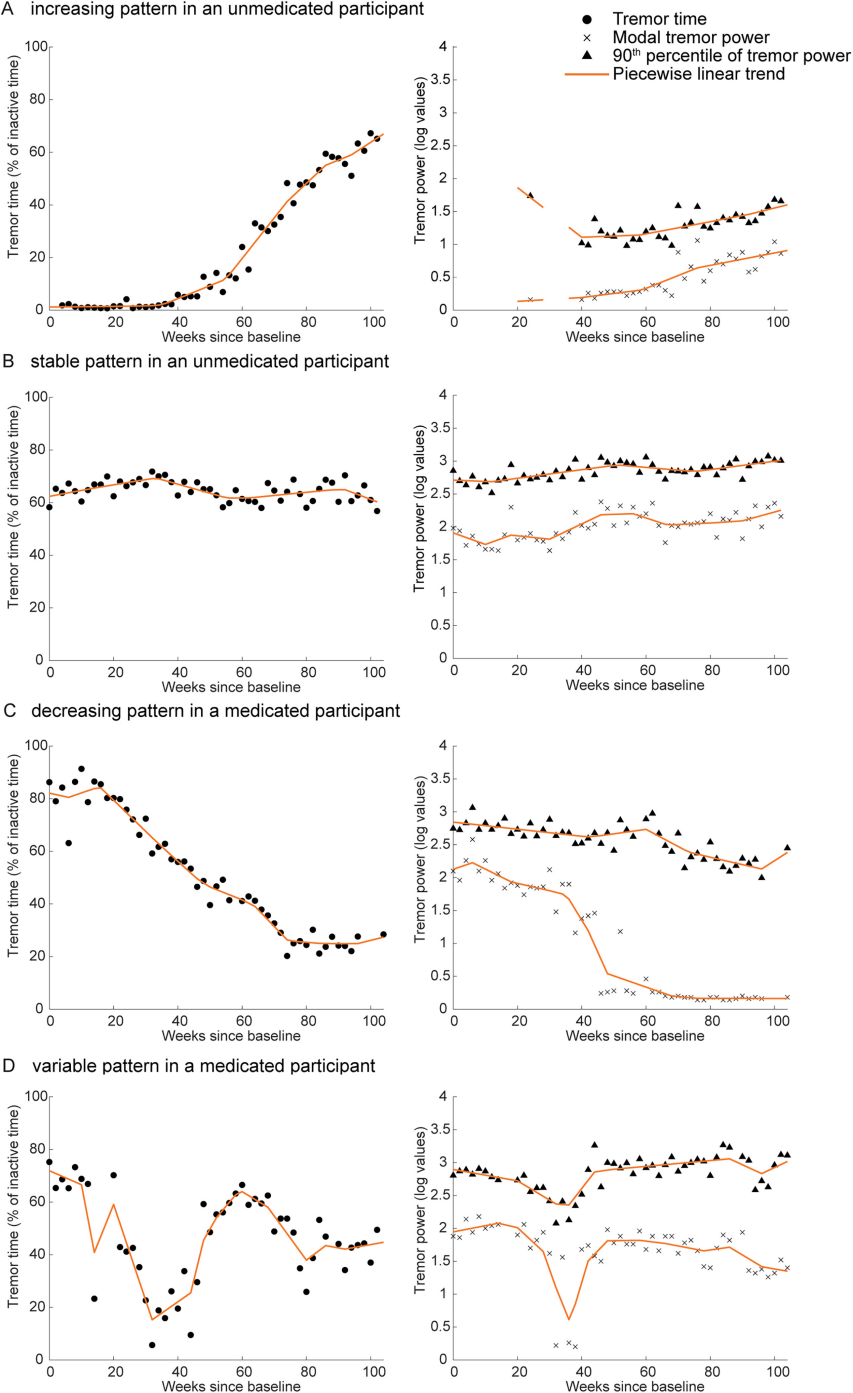
Examples of individual progression patterns in sensor‐derived tremor measures. Tremor time is shown on the left, and the modal and 90th percentile of tremor power in the graphs on the right. The 2 participants shown in A and B were not using symptomatic medication during the full study period, whereas the participants in C and D did. In A, no tremor power measures were derived for the first weeks, since tremor time was below the false positive threshold of 3.5% in those weeks. [Color figure can be viewed at www.annalsofneurology.org]

Tremor progression was defined as long‐term, within‐subject changes in tremor time or power. Figure 2 shows these changes at the group level, while Figure 3 provides examples of typical individual progression patterns. The distribution of absolute sensor‐derived tremor measures over time is given in Figure S3.

**FIGURE 4:**
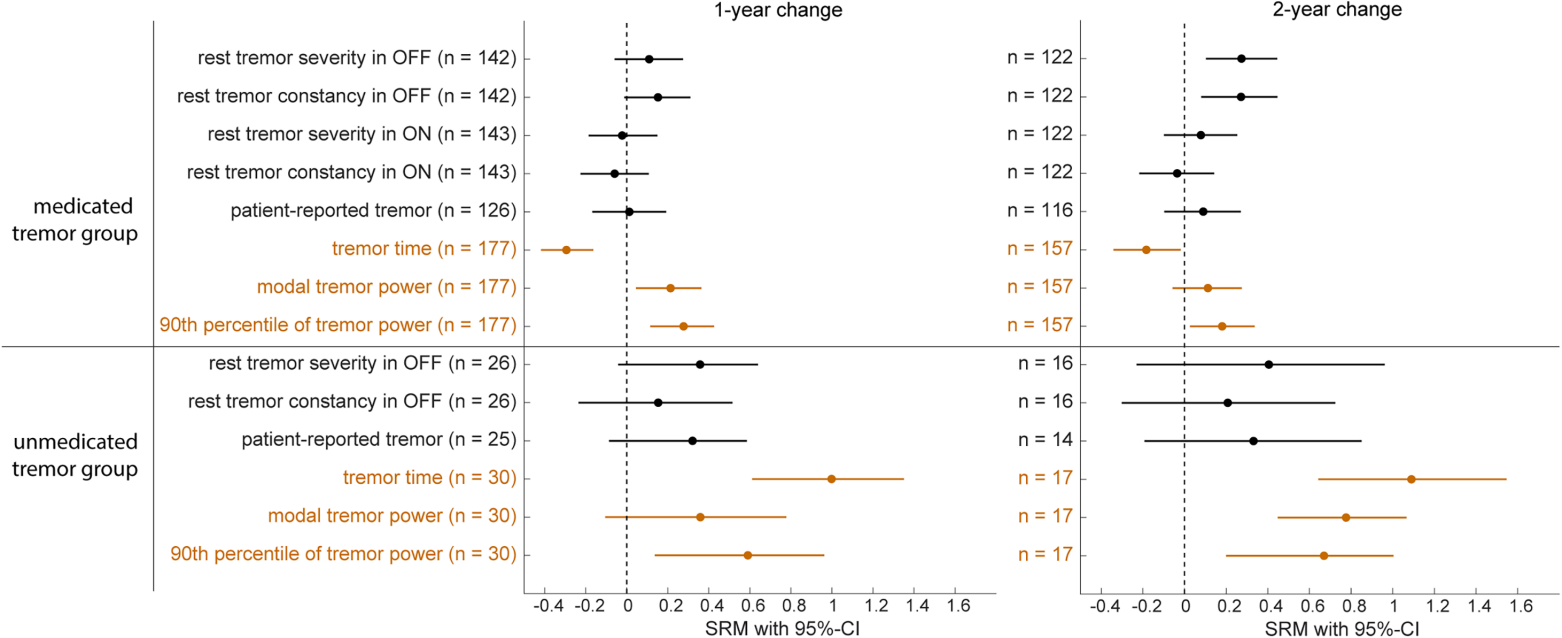
Standardized response mean (SRM) with 95% confidence interval (CI) of Movement Disorder Society‐Unified Parkinson's Disease Rating Scale (MDS‐UPDRS) tremor scores (black) and sensor‐derived tremor measures (orange) assessed at 1 and 2 years after baseline. All participants with tremor time above the false positive threshold at baseline and at 1‐ or 2‐year follow‐up were included. [Color figure can be viewed at www.annalsofneurology.org]

To compare the sensitivity to tremor progression between the sensor‐derived tremor measures and clinical tremor scores, we derived SRMs at 1‐ and 2‐year follow‐ups. In the unmedicated tremor group (ie, participants with tremor time above the false positive threshold), all sensor‐derived tremor measures increased over 2 years (Fig 4). We observed a large responsiveness of tremor time, already after 1 year. The modal and 90th percentile of tremor power demonstrated small‐to‐moderate responsiveness. In contrast to the sensitivity of sensor‐derived measures, we observed no or small responsiveness of MDS‐UPDRS tremor scores. The SRM for tremor time was significantly larger than those of all 3 MDS‐UPDRS tremor items at 1‐year follow‐up and significantly larger than the rest tremor constancy (MDS‐UPDRS 3.18) after 2 years (effect sizes ranged from 0.53 [95% CI, 0.11–1.00; *p* = 0.01]to 0.84 (95% CI 0.38–1.75; *p* = 0.002]; all differences and 95% CI are given in Table S3). The 90th percentile of tremor power also had a larger SRM at 1‐year follow‐up compared to all 3 MDS‐UPDRS tremor items (effect size ranged from 0.49 [95% CI, 0.04–0.93; *p* = 0.03] to 0.71 [95% CI, 0.23–1.21; *p* = 0.003]).

In the medicated tremor group, instead of a worsening of tremor time, we observed a small improvement in tremor time (Fig 4). In contrast, the modal and 90th percentile of tremor power slightly worsened. Regarding the sensitivity of clinical tremor scores, MDS‐UPDRS ON state and patient‐reported tremor scores did not show significant changes over time. Sensor‐derived tremor time was significantly more sensitive to improvement after 1 year compared to the clinical scores assessed in ON motor state and the patient‐reported tremor score (effect size ranged from −0.27 [95% CI, −0.48 to −0.06; *p* = 0.01] to −0.37 [95%‐CI, −0.60 to −0.13; *p* = 0.002]) (see Table S3). The MDS‐UPDRS OFF state assessments demonstrated slight worsening, comparable to the sensor‐derived tremor power measures. The findings for tremor time and clinical tremor ratings were similar in the full unmedicated and medicated groups (including participants with and without tremor) (see Fig S4).

### Sensitivity Analyses

To assess the impact of the follow‐up duration on SRMs for the sensor‐derived tremor measures in the 2‐year unmedicated (tremor) group, we visualized these over time in Figure S5. A significant worsening of tremor time was already observed after 30 weeks, but for the tremor power measures, this was only seen after 60 to 70 weeks. Figure S6 shows the SRMs based on the weekly tremor measures themselves (without estimating the piecewise linear trend) in the 2‐year unmedicated group. As expected, this assessment resulted in a more variable SRM over time. Although an increase in all 3 sensor‐derived tremor measures is still visible, this analysis shows that smoothing is important to isolate long‐term changes from weekly variability.

We adjusted for the effect of tremor on censoring because of treatment initiation by reweighting the unmedicated group using IPCW, but this only had a small effect on the obtained SRMs, without changing the overall conclusions (see Fig S7 for the unweighted results). Medicated participants who were censored from the 2‐year longitudinal analysis because of dropout were similar to those of the full baseline medicated group (Table S4).

Differences in tremor progression between the medicated and unmedicated groups could not be explained by differences in disease duration, age, and watch side (see Fig S8 for the SRMs obtained for the matched medicated group and Table S5 for the baseline characteristics of this group). Finally, we observed no differences in sensor‐derived tremor time related to watch‐sided hand dominance (Fig S9).

### Correlation with Patient‐Reported Changes

We observed small, but significant correlations with patient‐reported tremor severity (MDS‐UPDRS part II) for all sensor‐derived tremor measures (tremor time: ρ = 0.17 (*p* < 0.001; n = 375); modal tremor power: ρ = 0.17 (*p* < 0.05; n = 144); 90th percentile of tremor power: ρ = 0.19 (*p* < 0.05; n = 144). Regarding the clinical rest tremor scores, 2‐year changes in the rest tremor constancy score (MDS‐UPDRS 3.18) and patient‐reported tremor score were also weakly correlated, with Spearman's ρ of 0.18 (*p* < 0.001; n = 387) for the OFF score and 0.19 (*p* < 0.001; n = 336) for the ON score. However, changes in the patient‐reported tremor score did not significantly correlate with changes in the MDS‐UPDRS rest tremor severity scores, both in ON (Spearman's ρ of 0.04; *p* = 0.11; n = 336) and OFF (Spearman's ρ of 0.08; *p* = 0.42; n = 387).

### Effect of Changes in Dopaminergic Medication Dose

We assessed the association between 2‐year changes in dopaminergic medication dose and daily‐life tremor measures in the medicated group, adjusted for disease duration and watch side. A larger increase in LEDD over the 2‐year period was weakly associated with a greater reduction in tremor time (standardized β = −0.10; 95% CI, −0.21 – 0.01). In addition, a higher disease duration at baseline was associated with a greater reduction in tremor time (standardized β = −0.15; 95% CI, −0.26 to −0.03). Changes in the tremor power measures were not associated with changes in the studied clinical factors (see Fig S10).

**FIGURE 5:**
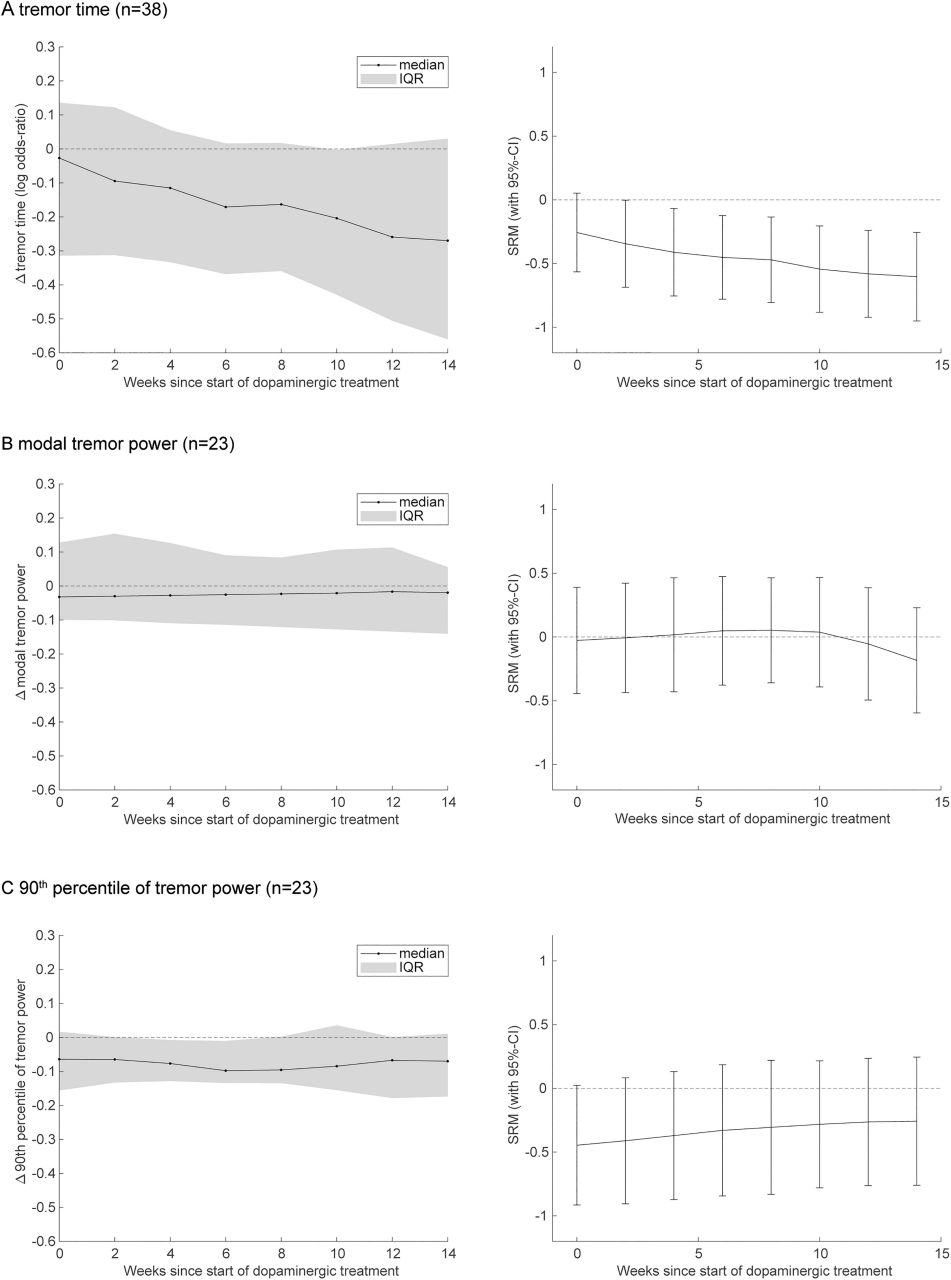
Responsiveness to the initiation of dopaminergic treatment of (A) tremor time, (B) modal tremor power, and (C) the 90th percentile of tremor power. The absolute changes in tremor measures are shown on the left (median and interquartal range [IQR]). From these changes, the standardized response means (SRMs) with 95% confidence intervals (CI) were computed, and shown on the right.

The responsiveness to dopaminergic treatment initiation of tremor time (n = 39) and of the modal and 90th percentile of tremor power (n = 23) are shown in Figure 5. Tremor time decreased after the initiation of treatment, whereas the tremor power measures did not show a significant decrease.

## Discussion

Using wrist sensor data obtained from 2 years of passive monitoring, we demonstrated that before symptomatic drug treatment initiation, daily‐life sensor‐derived measures are more sensitive to tremor progression than both clinician‐ and patient‐reported outcomes. In particular, sensor‐derived tremor time demonstrated a large responsiveness to tremor progression at both 1 and 2 years of follow‐up. By contrast, in medicated participants, tremor time significantly decreased over time, which was associated with both a longer disease duration and increasing dopaminergic medication dose (ie, LEDD). Together, these findings show that sensor‐derived tremor time is a sensitive digital biomarker for quantifying both tremor progression and treatment response, with promising applications in clinical trials and individual patient care.

Sensitive outcome measures are critical for quantifying disease progression in clinical trials, not only for PD, but also for other chronic diseases. Many new disease‐modifying therapies are being developed, but evaluating their efficacy currently requires large sample sizes (typically over 200 patients) and long follow‐up.^32^ In the unmedicated group, which is the target population for many disease‐modifying trials, tremor time already increased over 1 year of follow‐up, with an SRM approximately twice as large compared to those of MDS‐UPDRS rest tremor scores. Although tremor measures should be combined with measures of other PD symptoms in these trials, the results of this study are promising, because the required sample size of a typical randomized controlled trial design is directly related to the SRM of the outcome measure.^33^ The 1‐year SRM for tremor time that we observed in the full‐unmedicated group (0.65) is equal to that observed in the WATCH‐PD study.^7^ In unmedicated participants with tremor, we observed an even larger SRM (1.09 after 1 year), which was similar after 1 and 2 years of follow‐up.

In the medicated group, we observed a small, but significant improvement in tremor time, whereas the 90th percentile of tremor power slightly worsened over 1 and 2 years. Furthermore, a decrease in tremor time was associated with an increase in LEDD, whereas this association was absent for the 90th percentile of tremor power. Based on these findings, it is likely that the 90th percentile of tremor power reflects the tremor severity during OFF periods in daily life and is, therefore, more sensitive to capture disease progression in medicated participants. This aligns with findings from the clinical tremor assessments, showing that tremor severity and constancy in OFF also worsened over time in the medicated group, in agreement with previous studies.^17^, ^18^, ^19^ By contrast, weekly tremor time is likely more influenced by ON than OFF periods, since early‐stage PD participants on symptomatic medication typically spend most of their time in the ON state.^34^ Therefore, the observed improvements in tremor time can be partly explained by up‐titration of symptomatic medication in early‐stage PD. Noteworthy, improvements in tremor time were not only related to increases in LEDD in the medicated group, but also to increased disease duration. This aligns with previous findings that tremor increases in the early stages of PD but can disappear later in the disease (inverted u‐shape hypothesis of tremor progression^20^, ^35^, ^36^). This is important to consider in clinical trials, since the largest treatment effects are expected to be found in participants with short disease duration. In later disease stages and for non‐tremor dominant phenotypes, measuring other PD symptoms becomes more important.

Insights into individual daily‐life tremor time and power could also contribute to personalized treatment management in clinical care, by allowing for more informed decision‐making, accurate, and timely evaluation of treatment response. Because the response of tremor to dopaminergic medication highly varies between individuals, other treatment options including stress‐reducing strategies, non‐dopaminergic medication, or deep‐brain stimulation are also used.^37^, ^38^ Remote monitoring of the treatment response might allow for earlier treatment adjustments and ultimately improved quality of life for PD patients, but this remains to be elucidated in future research.^16^

An important requirement for using sensor‐derived measures in clinical trials and clinical care is the ability to capture meaningful changes.^9^, ^10^ Digital measures of tremor have been identified as relevant by persons with early PD,^39^ but showing the ability to capture meaningful changes remains difficult. Although we previously observed moderate correlations between sensor‐derived tremor measures and the patient‐reported tremor score cross‐sectionally,^23^ here, we observed small, but significant correlations longitudinally. This may be partly explained by the limited sensitivity to change of episodic patient‐reported tremor scores.^4^ A more suitable anchor to determine meaningful change might be the Patient Global Impression of Change scale (PGI‐C).^40^ By assessing both the PGI‐C score and the change in sensor‐derived tremor measures after an intervention where change is expected, the minimal clinically important differences of the tremor measures could be determined. Future research and collaboration with regulators will hopefully result in clearer recommendations for linking digital measures to meaningfulness.

### Strengths and Limitations

A key strength of this study is the use of a unique large dataset of continuous wrist sensor data, collected over a 2‐year period with minimal dropout and high wearing compliance.^24^ Second, in contrast to proprietary algorithms used in other studies, we applied a validated, open‐source, and device‐agnostic algorithm to derive daily‐life tremor measures, ensuring that our results are directly relevant for other studies collecting wrist gyroscope data. Third, we conducted in‐depth analyses regarding the performance of sensor‐derived tremor measures in both unmedicated and medicated groups of early PD. This could serve as an example for evaluating digital biomarkers in future research, for PD as well as for other chronic diseases. Finally, to obtain unbiased estimates of tremor progression in the unmedicated group, we corrected for potential informative censoring because of earlier treatment initiation in participants with more severe tremor. Our approach could be used in disease‐modifying clinical trials as well, which typically also censor patients after initiation of symptomatic treatment, but often do not account for informative censoring.^29^

A limitation of this study is that LEDD information was only yearly available, therefore, the effect of changes in LEDD on daily‐life tremor progression could not be assessed on a more granular week‐to‐week basis. Furthermore, the effect of dopaminergic treatment on tremor is highly variable, which could explain the weak negative relation between change in tremor time and change in LEDD.^37^ It is also possible that a more aggressive worsening of tremor over time necessitated a larger increase in LEDD, and the net result is a weak correlation between tremor worsening and LEDD because these 2 effects oppose each other. Next to this, we did not separately assess the effect of different non‐dopaminergic treatments on tremor. For our main analysis in the medicated group, we considered all symptomatic medication, including non‐dopaminergic medication. However, the proportion of non‐dopaminergic medication users was small (only 3% at baseline, see Table 1). Therefore, the effect of these types of treatment on the group‐level tremor progression observed in the medicated group is likely very small. Future research could assess the effect of non‐dopaminergic treatments on tremor progression as well. Another important consideration is that we focused only on rest (and re‐emergent) tremor. Although this is the most characteristic and prevalent tremor phenotype in PD, kinetic and pure postural tremor may have different progression patterns.^20^ Last, although the use of a 2‐year unmedicated group of PD participants is unique, the sample size of this subgroup was small because many participants initiated symptomatic therapy. The results of this study should, therefore, be replicated and validated in independent cohorts. Nevertheless, the findings on tremor progression in unmedicated PD are directly relevant to disease‐modifying clinical trials, as these focus increasingly on de novo PD or even prodromal disease stages.^41^ Tremor is the most common presenting symptom at disease onset and has been observed in prodromal cases.^35^, ^42^, ^43^, ^44^ Future research could explore whether real‐life detection of tremor in prodromal PD is possible as well.^45^ Furthermore, future work could assess whether the objectivity or granularity of sensor‐derived tremor measures contributes most to the enhanced sensitivity to change, by comparing in‐clinic tremor sensor measurements to continuous monitoring of tremor.

In conclusion, this study shows the potential of daily‐life sensor‐derived tremor measures for evaluating novel treatments in clinical trials. Our findings also contribute new insights into daily‐life tremor progression in PD. The sensor‐derived tremor measures could be combined with other biomarkers in future studies, such as neuroimaging markers or measures of stress, to improve understanding of individual variation in tremor expression. Last, although tremor is a highly relevant symptom for persons with early PD, other motor and non‐motor symptoms affect their quality of life as well.^12^ Future research should aim to combine real‐life monitoring of tremor with monitoring of other relevant symptoms in PD.

## Author Contributions

N.A.T., I.G.B., R.C.H., and L.J.W.E. contributed to the conception and design of the study; N.A.T., I.G.B., D.C.S., E.P., H.C., S.S., M.A.L., Y.P.R., B.R.B., R.C.H., and L.J.W.E. contributed to the acquisition and analysis of data; N.A.T. contributed to drafting the text and preparing the figures.

## Potential Conflicts of Interest

S.S. is currently employed by and holds shares in Verily Life Sciences, but declares no nonfinancial competing interests. All other authors declare no competing interests.

## Supporting information


**Table S1:** The optimal 𝜆 used for estimating the piecewise linear trends and subsequently derived proportion of variance explained by the fitted trends of sensor‐derived tremor measures. The variances were derived for medicated and unmedicated participants separately. IQR = interquartile range.
Table S2: Results of the Cox proportional hazards model fitted on participants unmedicated at baseline (*n = 78*), to estimate the effect of tremor on the time to treatment initiation. Different lags (4, 6 and 8 weeks) between the measurement of tremor time and the initiation of treatment were evaluated in separate models, since we expected that there is a delay between worsening of tremor and the moment of treatment initiation. The model with a lag of 4 weeks was selected based on the largest concordance statistic, although the difference between the three models was small.
Table S3: Differences (and 95% confidence intervals, assessed using bootstrapping) between standardized response means (SRMs) of sensor‐derived tremor measures and MDS‐UPDRS tremor scores, for the different subgroups and follow‐up periods used in the analyses. In the medicated groups, we compared the SRMs of tremor time to clinical rest tremor scores assessed in ON, whereas we compared the SRMs of the modal and 90th percentile of tremor power to those of OFF clinical scores. Significant differences are highlighted in bold.
Table S4: Baseline characteristics of medicated participants that were excluded from the two‐year analysis due to unavailability of sensor data at follow‐up, in addition to characteristics of the full baseline medicated group. IQR = inter‐quartile range. MDS‐UPDRS = Movement Disorder Society‐Sponsored Revision of the Unified Parkinson's Disease Rating Scale. Part 1: non‐motor experiences of daily living. Part 2: motor experiences of daily living. Part 3: motor examination. Part 4: motor complications
Table S5: Demographic and clinical characteristics, as well as sensor‐derived tremor measures at baseline of the unmedicated and matched medicated groups. The modal and 90th percentile of tremor power were assessed in all participants with tremor time above the false positive threshold at baseline (*n* = 47 for the unmedicated group and *n* = 38 for the matched medicated group). IQR = inter‐quartile range. MDS‐UPDRS = Movement Disorder Society‐Sponsored Revision of the Unified Parkinson's Disease Rating Scale. Part 1: non‐motor experiences of daily living. Part 2: motor experiences of daily living. Part 3: motor examination. Part 4: motor complications.
Figure S1: Number of unmedicated participants (with available sensor data in bold), which mainly decreased over time due to the initiation of symptomatic drug treatment.
Figure S2: Distribution of the two‐year clinical visit week numbers.
Figure S3: Distribution of sensor‐derived tremor measures over time in the medicated group (on the left) and unmedicated group (on the right). Tremor time (A) was derived for all participants and weeks in which data was available (upper plots), and subsequently in all participants and weeks in which tremor time was above the false positive threshold (lower plots). The modal (B) and 90th percentile of tremor power (C) were also assessed for the participants and weeks in which tremor time was above the false positive threshold. The number of included participants over time is shown in orange.
Figure S4: Standardized response mean (SRM) with 95% confidence interval (CI) of MDS‐UPDRS tremor scores (black) and sensor‐derived tremor measures (orange) assessed at one and two years after baseline. All participants with data available at baseline and at one‐ or two‐year follow‐up were included.
Figure S5: Standardized response mean (SRM) with 95% confidence interval (CI) of sensor‐derived tremor measures over time in the two‐year unmedicated group. Tremor time (A) was assessed in all participants with data available at baseline and at two‐year follow‐up (left), and subsequently in all participants with tremor time above the false positive threshold at baseline and two‐year follow‐up (right). The modal (B) and 90th percentile of tremor power (C) were also assessed in all participants with tremor time above the false positive threshold at baseline and at two‐year follow‐up.
Figure S6: Standardized response mean (SRM) with 95% confidence intervals (CI) of sensor‐derived tremor measures over time, based on weekly tremor measures assessed in the two‐year unmedicated group (without piecewise linear trend estimation). Tremor time (A) was assessed in all participants with data available at baseline and at two‐year follow‐up (on the left), and subsequently in all participants with tremor time above the false positive threshold at baseline and at two‐year follow‐up (on the right). The modal (B) and 90th percentile of tremor power (C) were also assessed in all participants with tremor time above the false positive threshold at baseline and at two‐year follow‐up.
Figure S7: Standardized response mean (SRM) with 95% confidence interval (CI) of MDS‐UPDRS tremor scores (black) and sensor‐derived tremor measures (orange) assessed at one and two years after baseline in the weighted and unweighted unmedicated (tremor) groups.
Figure S8: Standardized response mean (SRM) with 95% confidence interval (CI) of MDS‐UPDRS tremor scores (black) and sensor‐derived tremor measures (orange) assessed at one and two years after baseline, with and without matching the medicated group on the unmedicated group at baseline.
Figure S9: No differences in tremor time were found between participants wearing the watch on the dominant side and participants wearing it on the non‐dominant side, for both the medicated tremor group (A) and unmedicated tremor group (B). Differences were assessed using the Mann–Whitney *U*‐test.
Figure S10: (A) Shows the standardized β coefficients obtained by multivariable linear regression with two‐year changes in sensor‐derived tremor measures as individual outcomes and the following predictors: (1) changes in levodopa equivalent daily dose (LEDD), (2) disease duration at baseline and 3) watch side (more‐ or less‐affected side). In (B) the individual two‐year changes in tremor time against disease duration at baseline are shown, corrected for change in LEDD and taken as if the watch was worn on the more‐affected side.

## Data Availability

Data from the PPP used in the present study were retrieved from the PEP database (https://pep.cs.ru.nl/index.html). The PPP data is available on request via: ppp-data@radboudumc.nl. More details on the procedure can be found on the website www.personalizedparkinsonproject.com/home. The code used to conduct this study is publicly available at https://github.com/AI-for-Parkinson-Lab/longitudinal_tremor.
