# Supplementary material for: Daily‐Life, Sensor‐Derived Tremor Measures Are Sensitive to Progression in Early Parkinson's Disease
Source: Ann Neurol. 2026 May 12;100(2):282–94. doi: 10.1002/ana.78236 (PMC13387973; doi:10.1002/ana.78236)
Supplement: Supplementary file 1 — Table S1: The optimal 𝜆 used for estimating the piecewise linear trends and subsequently derived proportion of variance explained by the fitted trends of sensor‐derived tremor measures. The variances were derived for medicated and unmedicated participants separately. IQR = interquartile range. Table S2: Results of the Cox proportional hazards model fitted on participants unmedicated at baseline (n = 78), to estimate the effect of tremor on the time to treatment initiation. Different lags (4, 6 and 8 weeks) between the measurement of tremor time and the initiation of treatment were evaluated in separate models, since we expected that there is a delay between worsening of tremor and the moment of treatment initiation. The model with a lag of 4 weeks was selected based on the largest concordance statistic, although the difference between the three models was small. Table S3: Differences (and 95% confidence intervals, assessed using bootstrapping) between standardized response means (SRMs) of sensor‐derived tremor measures and MDS‐UPDRS tremor scores, for the different subgroups and follow‐up periods used in the analyses. In the medicated groups, we compared the SRMs of tremor time to clinical rest tremor scores assessed in ON, whereas we compared the SRMs of the modal and 90th percentile of tremor power to those of OFF clinical scores. Significant differences are highlighted in bold. Table S4: Baseline characteristics of medicated participants that were excluded from the two‐year analysis due to unavailability of sensor data at follow‐up, in addition to characteristics of the full baseline medicated group. IQR = inter‐quartile range. MDS‐UPDRS = Movement Disorder Society‐Sponsored Revision of the Unified Parkinson's Disease Rating Scale. Part 1: non‐motor experiences of daily living. Part 2: motor experiences of daily living. Part 3: motor examination. Part 4: motor complications Table S5: Demographic and clinical characteristics, as well as sensor‐derived tremor measu [file ANA-100-282-s001.pdf]

Supplementary material

Supplementary Table 1: The optimal λ used for estimating the piecewise linear trends and subsequently derived proportion of variance explained by the fitted trends of sensor-derived tremor measures. The variances were derived for medicated and unmedicated participants separately. IQR = interquartile range.

	λ	Proportion of variance explained by the fitted trends in medicated participants – median (IQR)	Proportion of variance explained by the fitted trends in unmedicated participants – median (IQR)
**Tremor time**	0.09	0.34 (0.15 – 0.61)	0.36 (0.12 – 0.69)
**Modal tremor power**	0.35	0.38 (0.21 – 0.62)	0.47 (0.25 – 0.60)
**90th percentile of tremor power**	0.56	0.43 (0.22 – 0.63)	0.43 (0.27 – 0.73)

Supplementary Table 2: Results of the Cox proportional hazards model fitted on participants unmedicated at baseline (n=78), to estimate the effect of tremor on the time to treatment initiation. Different lags (4, 6 and 8 weeks) between the measurement of tremor time and the initiation of treatment were evaluated in separate models, since we expected that there is a delay between worsening of tremor and the moment of treatment initiation. The model with a lag of 4 weeks was selected based on the largest concordance statistic, although the difference between the three models was small.

Lag between tremor time measurement week and initiation of treatment (number of weeks)	Beta coefficient in Cox proportional hazards model (95%-CI)	Concordance statistic
4	0.89 (-0.11 – 1.50)	0.535
6	0.83 (-0.17 – 1.51)	0.531
8	0.80 (-0.20 – 1.51)	0.528

Supplementary Table 3: Differences (and 95% confidence intervals, assessed using bootstrapping) between standardized response means (SRMs) of sensor-derived tremor measures and MDS-UPDRS tremor scores, for the different subgroups and follow-up periods used in the analyses. In the medicated groups, we compared the SRMs of tremor time to clinical rest tremor scores assessed in ON, whereas we compared the SRMs of the modal and 90th percentile of tremor power to those of OFF clinical scores. Significant differences are highlighted in bold.

			**Rest tremor severity**	**Rest tremor constancy**	**Patient-reported tremor**
Unmedicated tremor group	One-year change	Tremor time	**0.53 (0.11 – 1.00)**<br>n=26	**0.75 (0.29 – 1.31)**<br>n=26	**0.57 (0.13 – 1.19)**<br>n=25
		Modal tremor power	0.06 (-0.61 – 0.67)<br>n=26	0.28 (-0.22 – 0.83)<br>n=26	0.11 (-0.44 – 0.68)<br>n=25
		90th percentile of tremor power	**0.49 (0.04 – 0.93)**<br>n=26	**0.71 (0.23 – 1.21)**<br>n=26	**0.62 (0.22 – 1.12)**<br>n=25
	Two-year change	Tremor time	0.63 (-0.02 – 1.43)<br>n=16	**0.84 (0.38 – 1.75)**<br>n=16	0.70 (-0.19 – 1.70)<br>n=14
		Modal tremor power	0.33 (-0.44 – 0.95)<br>n=16	**0.53 (0.02 – 1.23)**<br>**n=16**	0.36 (-0.50 – 1.24)<br>n=14
		90th percentile of tremor power	0.26 (-0.39 – 1.06)<br>n=16	0.46 (-0.18 – 1.47)<br>n=16	0.56 (-0.03 – 1.47)<br>n=14
Unmedicated group	One-year change	Tremor time	0.20 (-0.07 – 0.59)<br>n=39	**0.49 (0.18 – 0.92)**<br>n=39	**0.56 (0.16 – 1.01)**<br>n=37
	Two-year change	Tremor time	0.14 (-0.40 – 0.68)<br>n=23	0.35 (-0.05 – 0.99)<br>n=23	**0.61 (0.02 – 1.29)**<br>n=19
Medicated tremor group	One-year change	Tremor time	**-0.31 (-0.52 - -0.10)**<br>n=143	**-0.27 (-0.48 - -0.06)**<br>n=143	**-0.37 (-0.60 - -0.13)**<br>n=126
		Modal tremor power	0.11 (-0.12 - 0.35)<br>n=142	0.06 (-0.16 - 0.30)<br>n=142	0.21 (-0.02 - 0.48)<br>n=126
		90th percentile of tremor power	0.18 (-0.03 - 0.41)<br>n=142	0.14 (-0.06 - 0.36)<br>n=142	**0.26 (0.04 - 0.51)**<br>n=126
	Two-year change	Tremor time	-0.22 (-0.46 – 0.02)<br>n=122	-0.11 (-0.32 – 0.10)<br>n=122	-0.24 (-0.49 – 0.01)<br>n=116
		Modal tremor power	-0.11 (-0.33 – 0.11)<br>n=122	-0.11 (-0.34 – 0.12)<br>n=122	0.05 (-0.19 – 0.29)<br>n=116
		90th percentile of tremor power	-0.09 (-0.30 – 0.12)<br>n=122	-0.09 (-0.32 – 0.14)<br>n=122	0.06 (-0.18 – 0.30)<br>n=116
Medicated group	One-year change	Tremor time	**-0.34 (-0.48 - -0.20)**<br>n=378	**-0.28 (-0.42 - -0.14)**<br>n=378	**-0.28 (-0.43 - -0.13)**<br>n=338
	Two-year change	Tremor time	**-0.25 (-0.39 - -0.11)**<br>n=357	-0.11 (-0.24 - 0.02)<br>n=357	-0.11 (-0.25 - 0.03)<br>n=329

Supplementary Table 4: Baseline characteristics of medicated participants that were excluded from the two-year analysis due to unavailability of sensor data at follow-up, in addition to characteristics of the full baseline medicated group. IQR: inter-quartile range. MDS-UPDRS: Movement Disorder Society-Sponsored Revision of the Unified Parkinson's Disease Rating Scale. Part 1: non-motor experiences of daily living. Part 2: motor experiences of daily living. Part 3: motor examination. Part 4: motor complications

		**Medicated participants excluded from the two-year follow-up analysis (n=30)**	**Medicated group (n=462)**
**Demographic and clinical characteristics at baseline**	Age (years), median (IQR)	62 (55 – 67)	62 (56 – 69)
	Gender (men), n (%)	18 (60)	273 (59)
	Disease duration (months), median (IQR)	33 (15 – 49)	33 (18 – 47)
	Watch worn on more-affected side, n (%)	15 (50)	246 (53)
	MDS-UPDRS part 1, median (IQR)	12 (6 – 17)	9 (7 – 14)
	MDS-UPDRS part 2, median (IQR)	8 (3 – 13)	7 (4 – 12)
	MDS-UPDRS part 3 OFF, median (IQR)	39 (28 – 47)	32 (24 – 42)
	MDS-UPDRS part 4, median (IQR)	4 (0 – 6)	2 (0 – 5)
	MDS-UPDRS part 3 OFF tremor subscore, median (IQR)	4 (0 – 11)	4 (1 – 7)
	Rest tremor severity device-sided arm, median (IQR)	0 (0 – 1)	0 (0 – 1)
	LEDD, median (IQR)	610 (400 – 810)	500 (300 – 688)
**Sensor-derived tremor measures at baseline**	Tremor time (%), median (IQR)	2.9 (1.0 – 8.0)	2.7 (1.2 – 8.4)

Supplementary Table 5: Demographic and clinical characteristics, as well as sensor-derived tremor measures at baseline of the unmedicated and matched medicated groups. The modal and 90th percentile of tremor power were assessed in all participants with tremor time above the false positive threshold at baseline (n=47 for the unmedicated group and n=38 for the matched medicated group). IQR: inter-quartile range. MDS-UPDRS: Movement Disorder Society-Sponsored Revision of the Unified Parkinson's Disease Rating Scale. Part 1: non-motor experiences of daily living. Part 2: motor experiences of daily living. Part 3: motor examination. Part 4: motor complications.

		**Unmedicated group (n=78)**	**Matched medicated group (n=78)**
**Demographic and clinical characteristics at baseline**	Age (years), median (IQR)	65 (56 – 70)	62 (55 – 69)
	Gender (men), n (%)	48 (62)	45 (58)
	Disease duration (months), median (IQR)	5 (2 – 13)	10 (7 – 14)
	Watch worn on more-affected side, n (%)	60 (78)	58 (74)
	MDS-UPDRS part 1, median (IQR)	5 (3 – 7)	9 (6 – 13)
	MDS-UPDRS part 2, median (IQR)	4 (3 – 8)	4 (2 – 9)
	MDS-UPDRS part 3 OFF, median (IQR)	34 (28 – 40)	28 (19 – 37)
	MDS-UPDRS part 4, median (IQR)	0 (0 – 1)	0 (0 – 4)
	MDS-UPDRS part 3 OFF tremor subscore, median (IQR)	5 (2 – 9)	4 (2 – 6)
	Rest tremor severity device-sided arm, median (IQR)	1 (0 – 2)	0 (0 -1)
**Sensor-derived tremor measures at baseline**	Tremor time (%), median (IQR)	5.8 (1.4 – 43.1)	2.8 (1.1 – 10.3)
	Modal tremor power (log values), median (IQR)	0.7 (0.2 – 1.2)	0.3 (0.2 – 0.5)
	90th percentile of tremor power (log values), median (IQR)	1.9 (1.5 – 2.3)	1.5 (1.2 – 1.9)

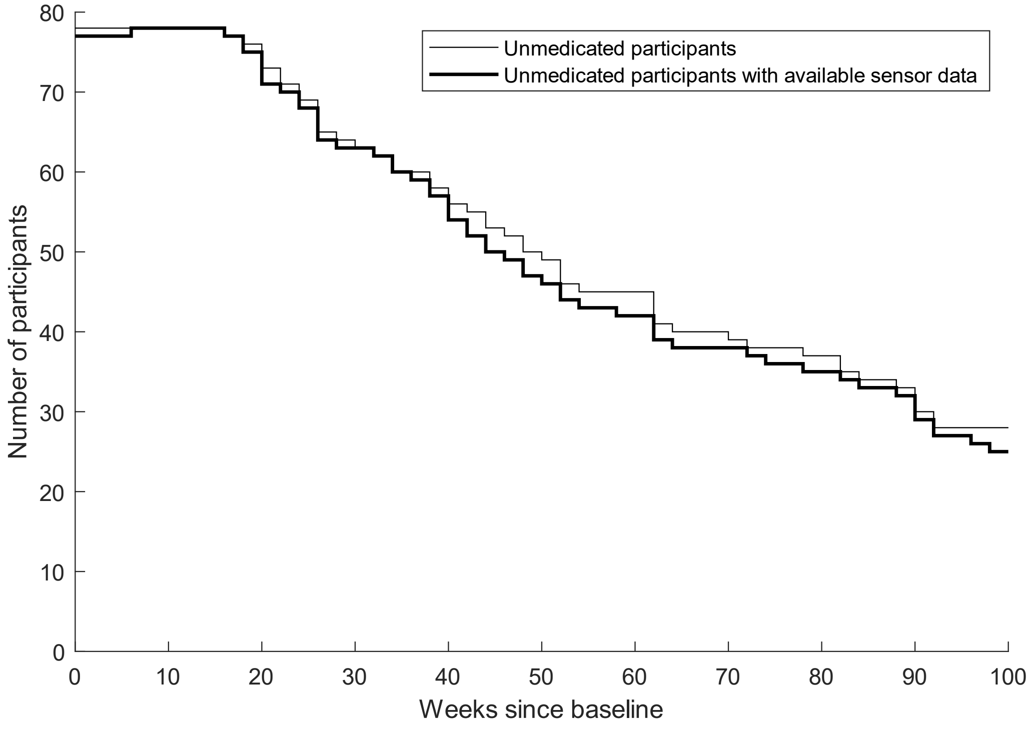

Supplementary Figure 1: Number of unmedicated participants (with available sensor data in bold), which mainly decreased over time due to the initiation of symptomatic drug treatment.

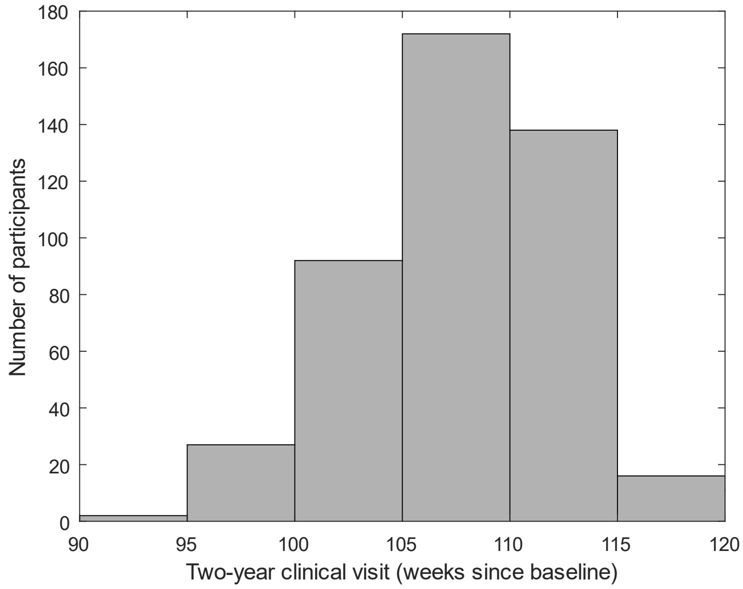

Supplementary Figure 2: Distribution of the two-year clinical visit week numbers.

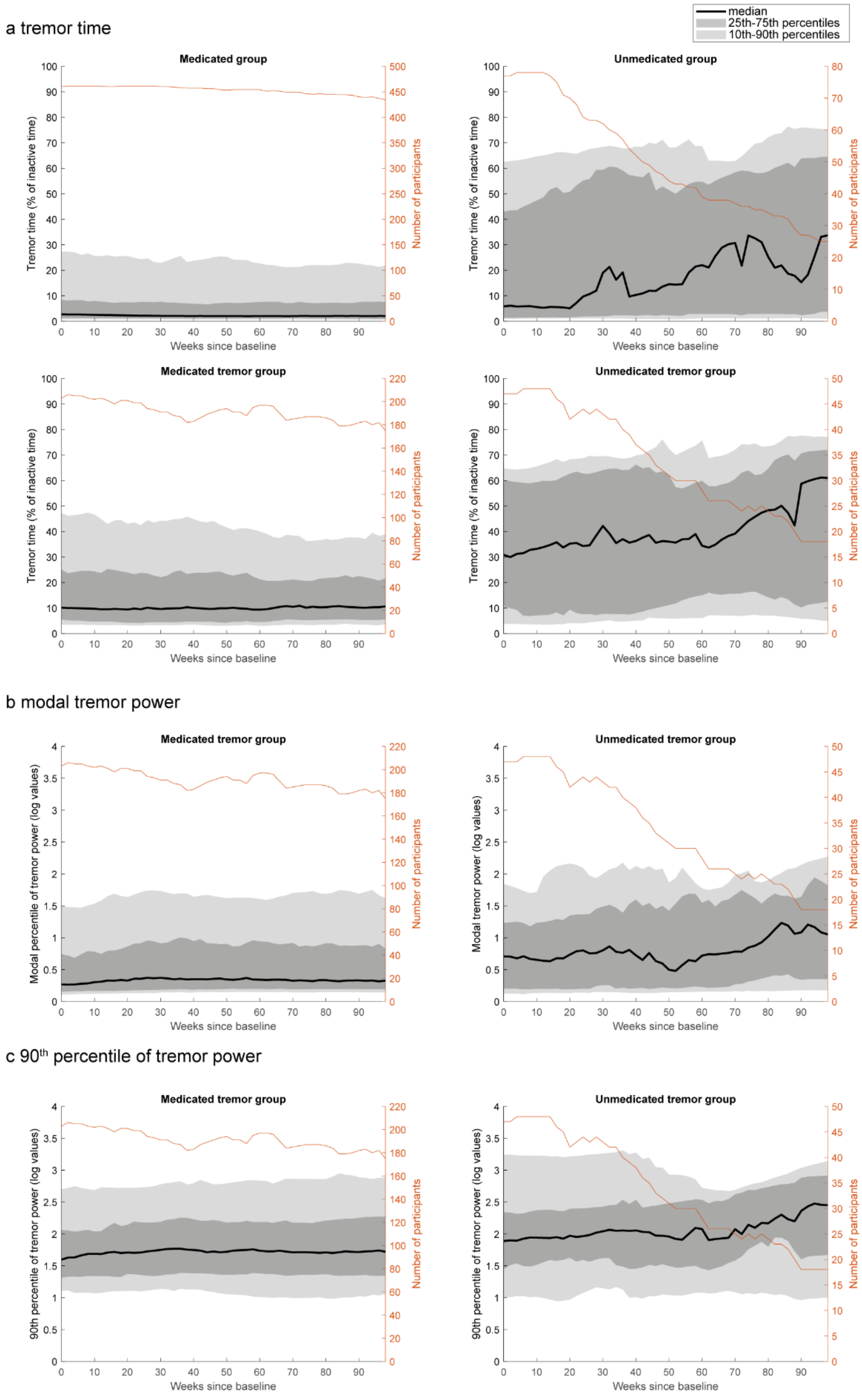

Supplementary Figure 3: Distribution of sensor-derived tremor measures over time in the medicated group (on the left) and unmedicated group (on the right). Tremor time (a) was derived for all participants and weeks in which data was available (upper plots), and subsequently in all participants and weeks in which tremor time was above the false positive threshold (lower plots). The modal (b) and 90th percentile of tremor power (c) were also assessed for the participants and weeks in which tremor time was above the false positive threshold. The number of included participants over time is shown in orange.

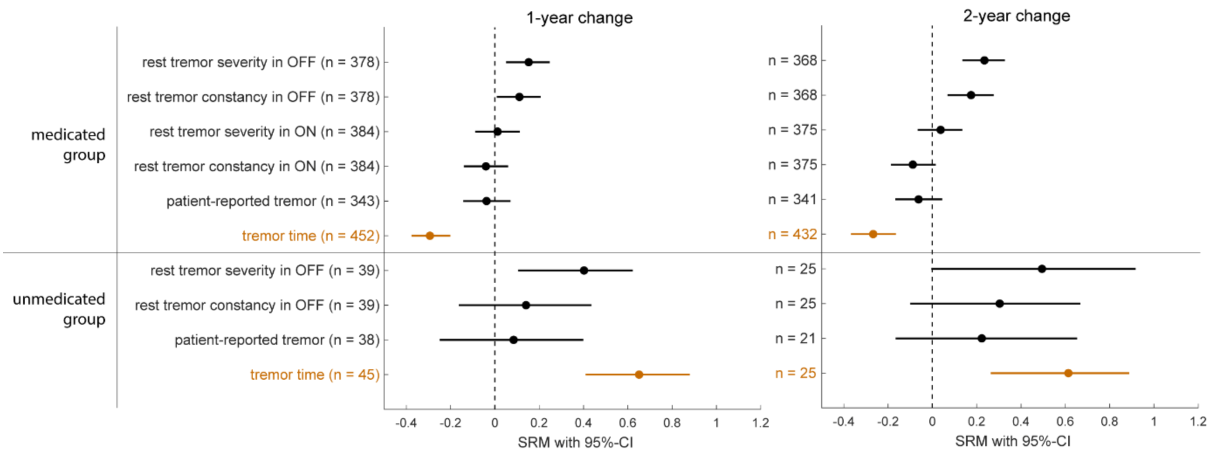

Supplementary Figure 4: Standardized response mean (SRM) with 95% confidence interval (CI) of MDS-UPDRS tremor scores (black) and sensor-derived tremor measures (orange) assessed at one and two years after baseline. All participants with data available at baseline and at one- or two-year follow-up were included.

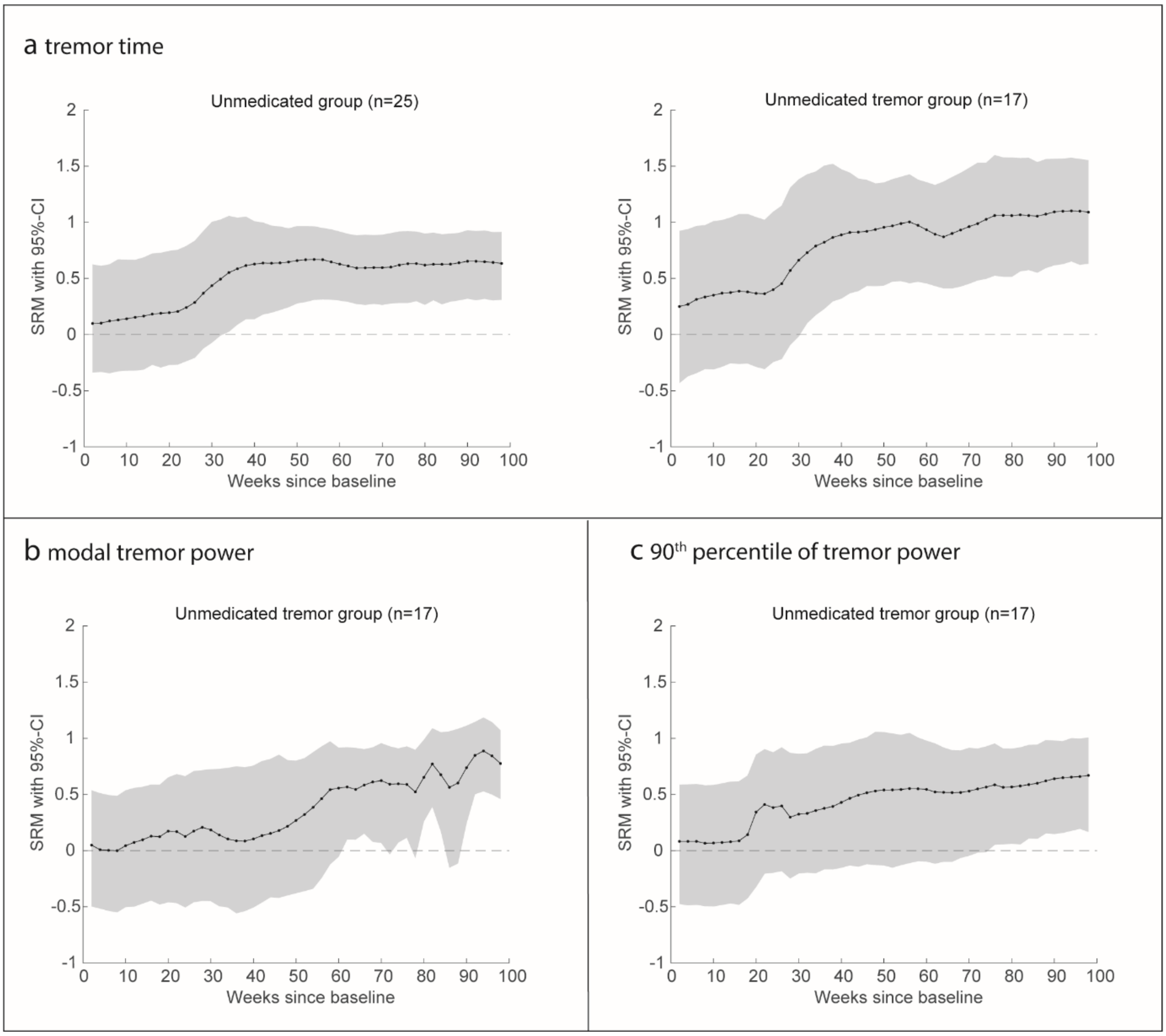

Supplementary Figure 5: Standardized response mean (SRM) with 95% confidence interval (CI) of sensor-derived tremor measures over time in the two-year unmedicated group. Tremor time (a) was assessed in all participants with data available at baseline and at two-year follow-up (left), and subsequently in all participants with tremor time above the false positive threshold at baseline and two-year follow-up (right). The modal (b) and 90th percentile of tremor power (c) were also assessed in all participants with tremor time above the false positive threshold at baseline and at two-year follow-up.

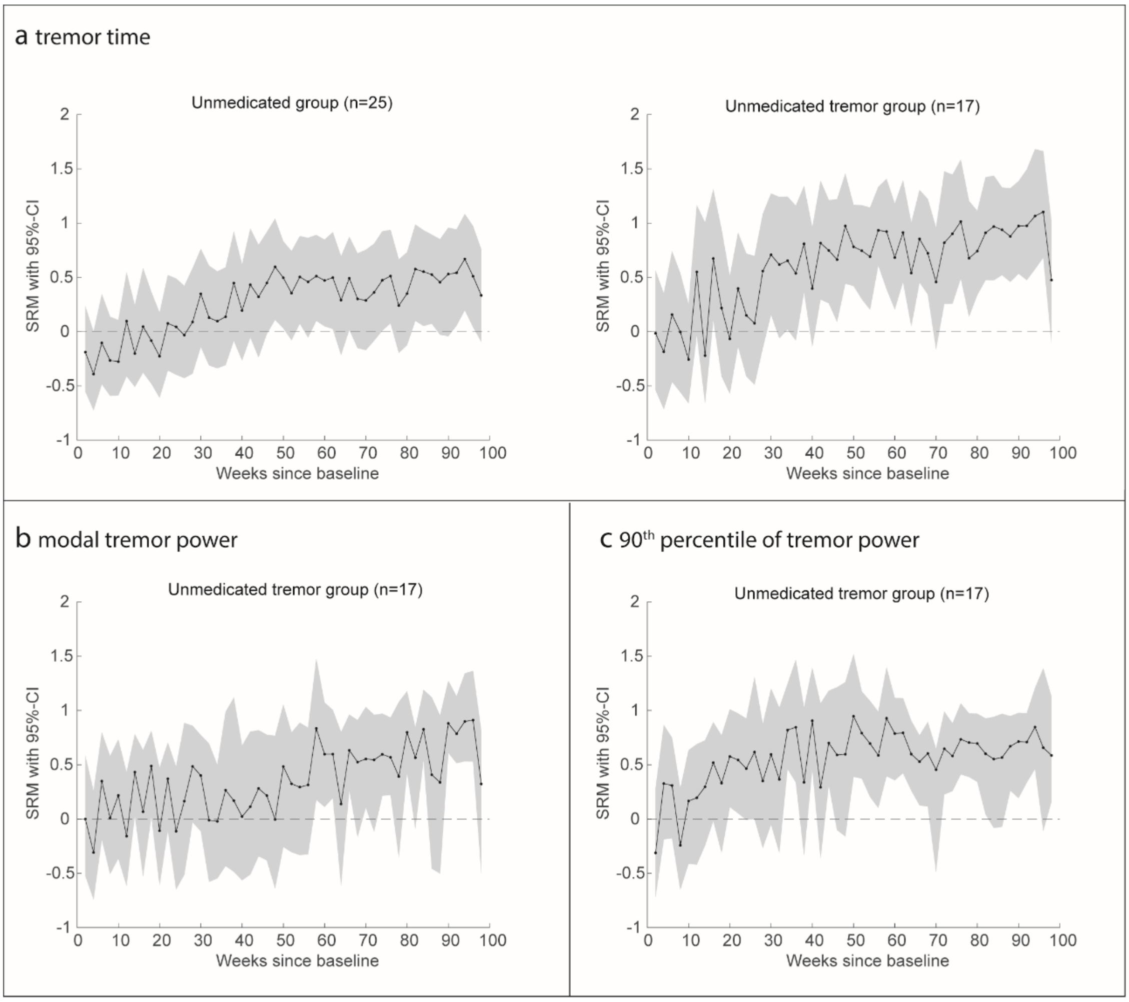

Supplementary Figure 6: Standardized response mean (SRM) with 95% confidence intervals (CI) of sensor-derived tremor measures over time, based on weekly tremor measures assessed in the two-year unmedicated group (without piecewise linear trend estimation). Tremor time (a) was assessed in all participants with data available at baseline and at two-year follow-up (on the left), and subsequently in all participants with tremor time above the false positive threshold at baseline and at two-year follow-up (on the right). The modal (b) and 90th percentile of tremor power (c) were also assessed in all participants with tremor time above the false positive threshold at baseline and at two-year follow-up.

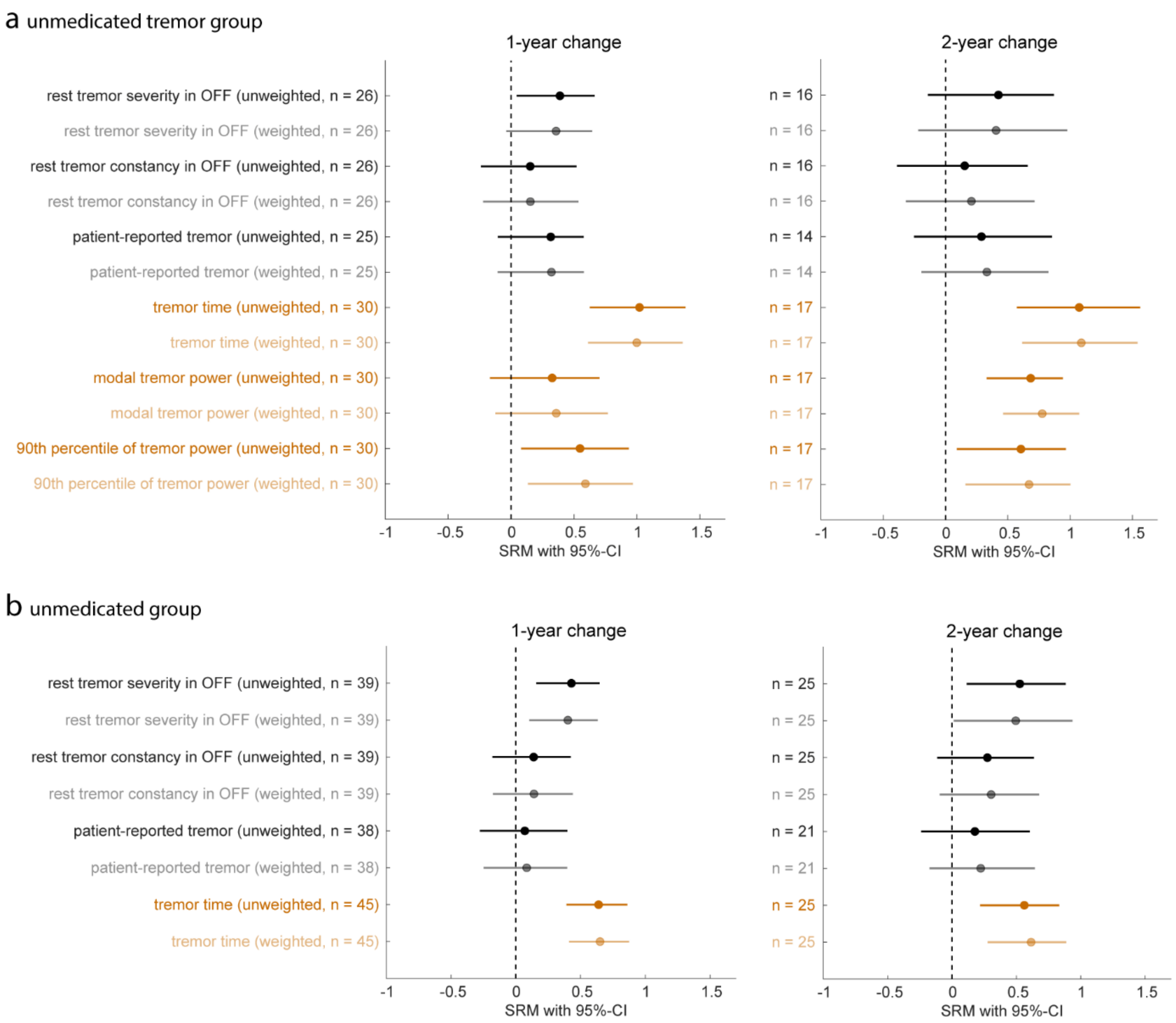

Supplementary Figure 7: Standardized response mean (SRM) with 95% confidence interval (CI) of MDS-UPDRS tremor scores (black) and sensor-derived tremor measures (orange) assessed at one and two years after baseline in the weighted and unweighted unmedicated (tremor) groups.

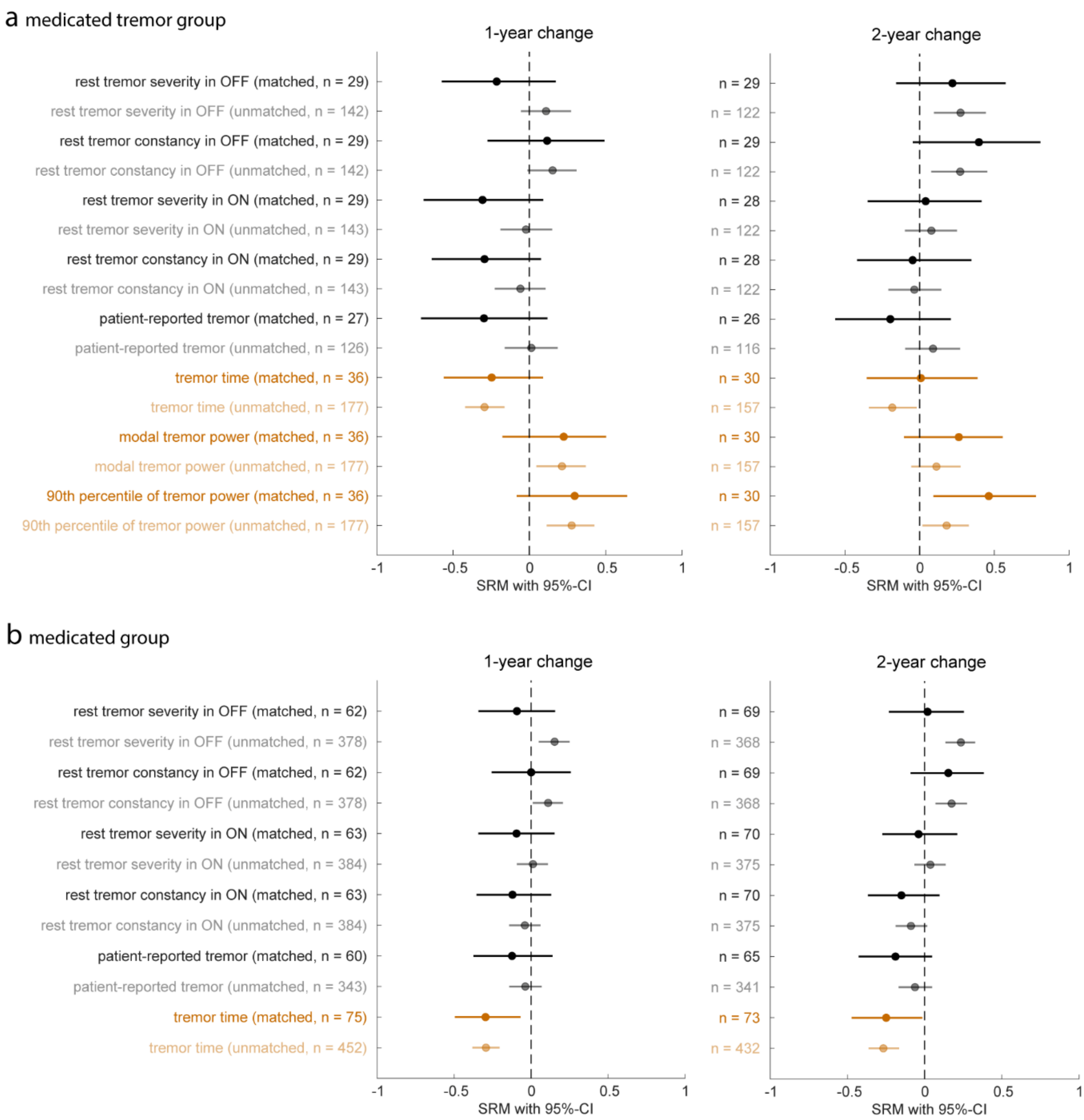

Supplementary Figure 8: Standardized response mean (SRM) with 95% confidence interval (CI) of MDS-UPDRS tremor scores (black) and sensor-derived tremor measures (orange) assessed at one and two years after baseline, with and without matching the medicated group on the unmedicated group at baseline.

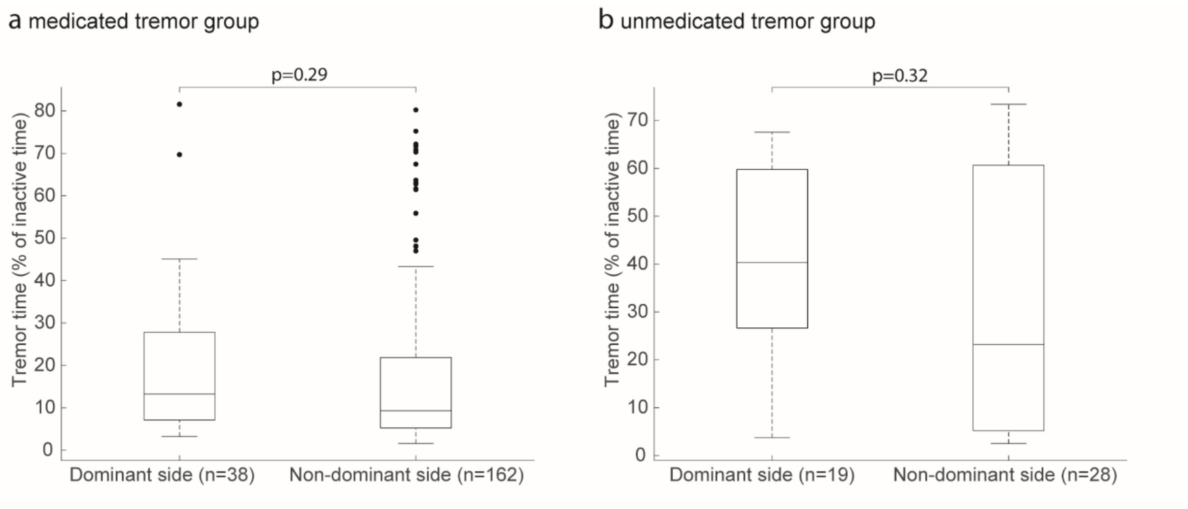

Supplementary Figure 9: No differences in tremor time were found between participants wearing the watch on the dominant side and participants wearing it on the non-dominant side, for both the medicated tremor group (a) and unmedicated tremor group (b). Differences were assessed using the Mann-Whitney U-test.

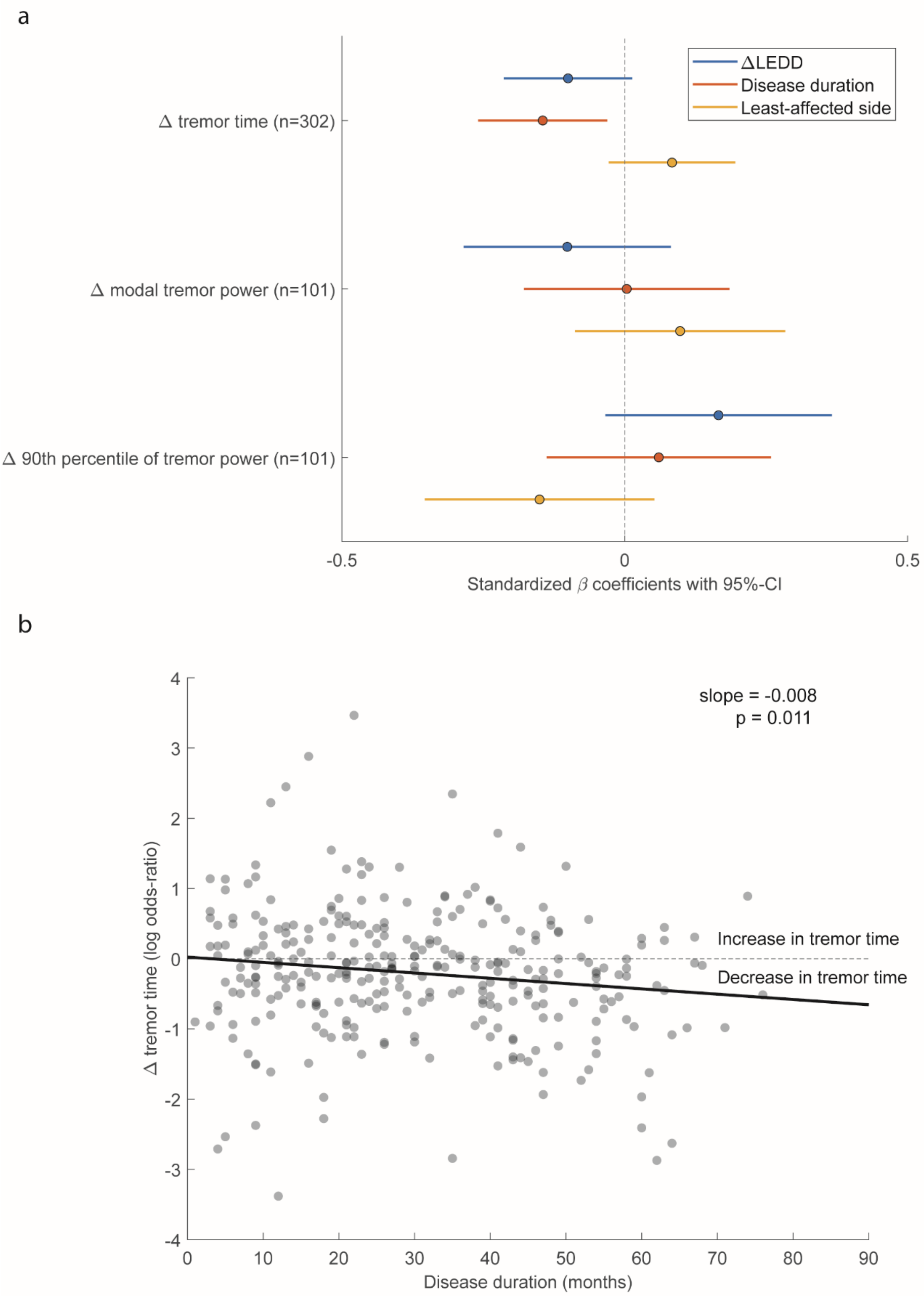

*Supplementary Figure 10: **a** shows the standardized β coefficients obtained by multivariable linear regression with two-year changes in sensor-derived tremor measures as individual outcomes and the following predictors: 1) changes in levodopa equivalent daily dose (LEDD), 2) disease duration at baseline and 3) watch side (more- or less-affected side). In **b** the individual two-year changes in tremor time against disease duration at baseline are shown, corrected for change in LEDD and taken as if the watch was worn on the more-affected side.*
